# Effects of Compound Danshen Injection Combined with Magnesium Sulfate on Pregnancy-Induced Hypertension Syndrome under the Guidance of Empirical Mode Decomposition Algorithm-Based Ultrasound Image

**DOI:** 10.1155/2021/9026223

**Published:** 2021-10-25

**Authors:** Xia Zhao, Hongbin Wang, Yanan Gao, Yanan Wang

**Affiliations:** Department of Obstetrics and Gynecology, The Fourth Hospital of Shijiazhuang, Shijiazhuang 050011, China

## Abstract

**Objective:**

The study focused on the separation effects of ultrasound blood flow signal detection, based on empirical mode decomposition (EMD) algorithm, and the clinical efficacy of Compound Danshen injection and magnesium sulfate in the treatment of pregnancy-induced hypertension (PIH) syndrome.

**Methods:**

The empirical mode decomposition (EMD) algorithm was optimized first and compared with other algorithms for the accuracy and stability in separation of blood flow signals. 80 patients with PIH syndrome undergoing ultrasound examination were selected as the research subjects and randomly divided into control group and observation group according to the actual treatment methods. 40 cases in the observation group were treated with Compound Danshen injection + magnesium sulfate, and 40 cases in the control group were treated with magnesium sulfate. After the treatment, the clinical indicators of the two groups of patients were analyzed.

**Results:**

The accuracy and stability in separating blood flow signal of the optimized EMD algorithm were better than those of other algorithms. After treatment, the total effective rate and blood pressure control of the observation group were significantly better than those of the control group, and the incidence of adverse maternal and infant outcomes was significantly lower than that of the control group. After treatment, the endothelin-1 (ET-1), C-reactive protein (CRP), and homocysteine (Hcy) indexes of the two groups of patients decreased significantly, and the decrease level of the observation group was significantly greater than that of the control group (*P* *<* 0.05). The prothrombin time (PT), fibrinogen (FIB), activated partial thromboplastin time (APTT), and plasma thrombin time (TT) levels of the two groups after treatment were better than those before treatment, and the observation group was better than the control group (*P* *<* 0.05).

**Conclusion:**

The optimized EMD algorithm is of great value for the separation of ultrasound blood flow signals. For patients with PIH syndrome, Compound Danshen injection combined with magnesium sulfate can be used as a treatment plan, which can improve maternal and infant outcomes; control blood pressure; reduce 24 h urine protein and serum ET-1, Hcy, and CRP levels; and improve coagulation function. It is worthy of promotion.

## 1. Introduction

Usually, pregnancy-induced hypertension (PIH) syndrome occurs in women with more than 20 weeks of pregnancy. Clinically, it mainly manifests itself as hypertension, proteinuria, and edema of limbs [1, 2]. PIH syndrome threatens maternal and infant health and can easily cause adverse events such as premature delivery, eclampsia, postpartum infection, or hemorrhage, which is not conducive to pregnancy outcome [3, 4]. Currently, the etiology of hypertension in pregnant women is mainly summarized as systemic small vasospasm [5]. Therefore, in practice, it is necessary to relieve systemic small vasospasm, and this strategy has been proved to have a significant effect [6]. Magnesium sulfate is a clinically effective drug for relieving systemic small vasospasm, and it is found to be effective in the treatment of hypertension in pregnancy [7]. However, the use of magnesium sulfate alone cannot achieve satisfactory results. Compound Danshen injection is a Chinese patent medicine mainly composed of Danshen (a traditional Chinese medicine to promote blood circulation and dredge meridians) and Jiangxiang (a traditional Chinese medicine to remove stasis and stop bleeding) [8]. Compound Danshen injection has a variety of pharmacological effects, such as expanding blood vessels, improving microcirculation, scavenging oxygen free radical, protecting liver function, and sedating the mind [9], which can effectively lower blood pressure, thereby alleviating vascular endothelial injury [10]. Generally speaking, to treat small vasospasm in patients is to restore the secretion function of nitric oxide and vascular endothelin [11]. Research has found that Danshen has a good effect on improving this function [12].

Ultrasound Doppler technology has become an important method for the diagnosis and efficacy evaluation of diseases of the blood circulatory system, especially cardiovascular diseases, because of its ability to measure blood flow nondestructively. Due to the simplicity and noninvasiveness of ultrasound diagnosis technology, in recent years, ultrasound examination has gradually been used in the functional and morphological evaluation of PIH. Studies have shown that functional and morphological changes (such as endothelial diastolic function and vascular stiffness) are related to the occurrence and development of PIH. However, the blood flow signal detected by traditional ultrasound often contains the signal of the tube wall, which will interfere with the blood flow speed to a certain extent, leading to deviations in the diagnosis and evaluation of the disease. Empirical mode decomposition (EMD) has significant advantages in the processing of nonlinear and nonstationary signals. However, it adds the first few layers as the blood flow signal during the separation process, which leads to a certain deviation in the separation results. Hence, it needs to be further optimized.

In this study, the EMD algorithm was optimized to separate ultrasound Doppler blood flow and vessel wall signals. 80 patients with PIH syndrome were taken as the research subjects to explore the effect of the combined therapy of Compound Danshen injection and magnesium sulfate on PIH, expected to provide reference value for its clinical application.

## 2. Research Methods

### 2.1. Research Subjects and Grouping

80 patients with PIH syndrome admitted to the hospital from July 2019 to August 2020 were selected as research subjects, and they were divided into a control group and an observation group according to different treatments. There were 40 cases in the control group, aged 23–40 years, with an average age of 29.34 ± 7.56 years. The gestational age was 31–41 weeks, with an average gestational age of 34.86 ± 3.95 weeks. There were 31 primiparous women and 9 postpartum women. There were also 40 cases in the observation group, aged 22–40 years, with an average age of 28.55 ± 6.94 years. The gestational age was between 32–41 weeks, with an average gestational age of 35.62 ± 3.80 weeks. There were 32 primiparous women and 8 postpartum women. The inclusion criteria of this study were as follows: (1) meeting the diagnosis criteria for the pregnancy-induced hypertension syndrome; (2) single pregnancy. Patients with other serious gynecological diseases were excluded. The experimental procedure of this study has been approved by the ethics committee of the hospital, and all the subjects included in the study have signed informed consent form. The two groups were compared for the age, gestational age, number of pregnancies, and other data. A *P* value greater than 0.05 was considered comparable.

### 2.2. The Separation Method of Blood Flow and Tube Wall Signals Based on EMD Algorithm

The blood flow is the integration of multiple moving blood cells, and the ultrasound Doppler signal scattered back by a single blood particle can be expressed as follows:

(1) $$\begin{array}{c} S_{i}\left(t\right)=A_{i}B_{i}\cos\left[\varphi \left(t\right)+\psi_{i}\right]C_{i}^{t}\left(t\right)C_{i}^{r}\left(t\right), \end{array}$$

where *i* represents the number of the particle; *A*_*i*_ indicates the acoustic field characteristics of the particle's location; *B*_*i*_ is the scattering coefficient of the particle.

*C*_*i*_^*t*^(*t*) and *C*_*i*_^*r*^(*t*) represent the shapes of the ultrasound transmitting gate and receiving gate, respectively; and *φ*(*t*) is the particle motion coefficient, *φ*(*t*)=*ω*_0_*t*+*φ*_*d*_(*t*), where *ω*_0_ is the angular frequency of ultrasonic emission and *φ*_*d*_(*t*) represents the phase change caused by the motion of the particle.

Assuming that the blood vessel is a straight round tube, the radial distribution of blood flow velocity can be expressed as follows.

(2) $$\begin{array}{c} s\left(y,t\right)=2v_{0}\left(1-y^{2}\right)+\sum_{p}v_{p}{\left|\psi \right|}_{p}\cos\left(\mathrm{pwt}+\varepsilon_{p}+\chi_{p}\right), \end{array}$$

where

(3) $$\begin{array}{c} \psi =\alpha i^{3/2}\frac{J_{0}\left(\alpha i^{3/2}\right)-J_{0}\left(y\alpha i^{3/2}\right)}{\alpha i^{3/2}J_{0}\left(\alpha i^{3/2}\right)-2J_{1}\left(\alpha i^{3/2}\right)}, \end{array}$$

where *y* is the ratio of the distance from the center of the blood vessel to the radius of the blood vessel; *J*_0_ and *J*_1_ represent the Bessel function of the orders of 0 and 1, respectively; *v*_*p*_represents the magnitude of the p-order Fourier coefficient of the average blood flow velocity curve; *ε*_*p*_ represents the phase of the p-order Fourier coefficient of the average blood flow velocity curve; *w* is the angular frequency of the heart beat; *χ* is the phase of *ψ*; and *v*_0_ is the initial blood flow velocity.

The pulsation of the blood vessel wall can be analyzed by the curve of blood pressure over time. Assuming that the cross-sectional area of the blood vessel is *S* and the blood pressure is *P*, the characteristic function of the blood vessel wall can be expressed as follows:

(4) $$\begin{array}{c} S\left(t\right)=ae^{bP\left(t\right)}+c, \end{array}$$

where *a*, *b*, *e* are constants related to the physiological characteristics of the blood vessel wall:*a*=3.6*cm*^2^, *b*=−0.0119*mmHg*, *c*=3.05*cm*^2^.

Then, the pulsation velocity curve of the blood vessel wall can be expressed as follows:

(5) $$\begin{array}{c} v\left(t\right)=\frac{dr\left(t\right)}{dt}, \end{array}$$

where *r*(*t*) is the radius of the blood vessel, $r\left(t\right)=\sqrt{S\left(t\right)/\pi }$.

According to the Doppler frequency shift formula, the ultrasonic Doppler tube wall signal is calculated as follows:

(6) $$\begin{array}{c} x_{w}=a_{w}\exp\left[i\phi_{d}\left(t\right)\right], \end{array}$$

where *a*_*w*_ represents the constant of the signal amplitude of the tube wall.

The original signal is decomposed into the eigenmode function (IMF) of each layer through the EMD algorithm, and the original signal is finally *N* − 1 decomposed into *N* − 1 IMF and a residual component.

(7) $$\begin{array}{c} s\left(t\right)=\sum_{t=1}^{N-1}x_{i}\left(t\right)+r\left(t\right), \end{array}$$

where *r*(*t*) is the residual component.

For further time-frequency analysis of the original signal, the Hilbert transform needs to be performed on the IMF decomposed by the EMD algorithm. Then, the Hilbert transform of *x*_*i*_(*t*) can be expressed as follows:

(8) $$\begin{array}{c} y_{i}\left(t\right)=\frac{1}{\pi }\int_{-\infty }^{+\infty }\frac{x_{i}\left(\tau \right)}{t-\tau }d\tau . \end{array}$$

The analytical signal *z*_*i*_(*t*) of *x*_*i*_(*t*) can be expressed as follows:

(9) $$\begin{array}{c} z_{i}\left(t\right)=x_{i}\left(t\right)+iy_{i}\left(t\right)=a_{i}\left(t\right)e^{\beta_{i}\left(t\right)}, \end{array}$$

where *a*_*i*_(*t*) represents the amplitude of the analytical signal, $a_{i}\left(t\right)=\sqrt{x_{i}^{2}\left(t\right)-y_{i}^{2}\left(t\right)}$; and *β*_*i*_(*t*) represents the phase angle of the analytical signal, *β*_*i*_(*t*)=arctan[*y*_*i*_(*t*)/*x*_*i*_(*t*)].

The instantaneous frequency is calculated as follows:

(10) $$\begin{array}{c} w_{i}\left(t\right)=\frac{d\beta_{i}\left(t\right)}{dt}. \end{array}$$

Then, the original blood flow signal can be expressed as follows:

(11) st=Re∑i=1naitexpi∫witdt.

In order to improve the accuracy of extracting pipe wall signals, an adaptive wavelet threshold algorithm is introduced to optimize the EMD algorithm. The *i*-th layer IMF component *x*_*i*_(*t*) can be expressed as follows:

(12) $$\begin{array}{c} x_{i}\left(t\right)=\left\{\begin{array}{cc} b_{i}\left(t\right) & 1\leq i\leq k-1\\ b_{i}\left(t\right)+w_{i}\left(t\right) & k\leq i\leq M-1\\ w_{i}\left(t\right) & M\leq i\leq N \end{array}\right., \end{array}$$

where *b*_*i*_(*t*) is the blood flow signal; *w*_*i*_(*t*) is the wall signal; and *k*, *M*, and *N* are the numbers of different layers of the IMF component: *k* is the number of the blood flow signal layers, *M* is the number of layers of IMF component containing both the tube wall and the blood flow signal, and *N* is the number of IMF component layers of the tube wall signal.

According to the noise power estimation algorithm in wavelet decomposition and the characteristics of EMD decomposition, the power of the first layer blood flow signal IMF is expressed as follows:

(13) $$\begin{array}{c} \sigma_{1}=\frac{\mathrm{median}\left\{\left|x_{1}\left(t\right)-\mathrm{median}x_{1}\left(t\right)\right|\right\}}{0.6745}. \end{array}$$

The power of the blood flow signal IMF in the *i*-th layer can be expressed as follows.

(14) $$\begin{array}{c} \sigma_{i}=\frac{\sigma_{1}}{{\sqrt{2}}^{i-1}}. \end{array}$$

The threshold denoising method is used to remove the blood flow signal in the tube wall signal, and the tube wall signal can be expressed as follows:

(15) $$\begin{array}{c} w_{i}=\left\{\begin{array}{cc} \mathrm{sgn}\left[x_{i}\left(t\right)\right]\left\{\left|x_{i}\left(t\right)\right|-\frac{2\sigma_{i}}{1+\exp\left\{L\left[x_{i}^{2}\left(t\right)-\sigma_{i}^{2}\right]\right\}}\right\} & \left|x_{i}\left(t\right)\right|>\sigma_{i}\\ 0 & \left|x_{i}\left(t\right)\right|\leq \sigma_{i}. \end{array}\right. \end{array}$$

Finally, the signal is reconstructed, and the signal calculation method of the original pipe wall is as follows:

(16) $$\begin{array}{c} w\left(t\right)=\sum_{i=k}^{M-1}w_{i}\left(t\right)+\sum_{i=M}^{n}x_{i}\left(t\right). \end{array}$$

**Figure 1:**
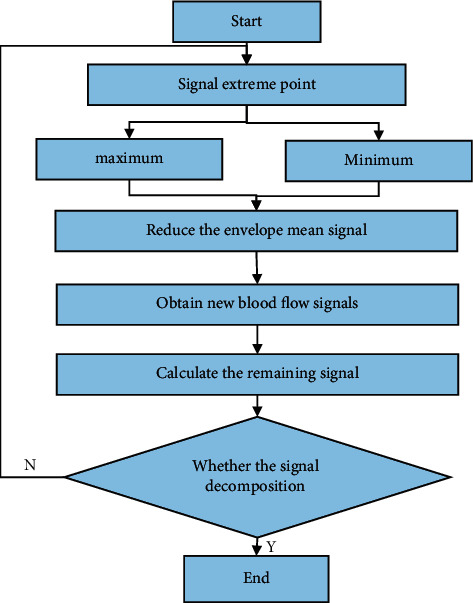
Flow chart of blood flow signal separation by the optimized EMD algorithm.

Then, the extreme values of the original ultrasonic blood flow signal are solved, then the upper and lower envelopes are obtained, and the average value of the upper and lower envelopes is subtracted from the initial signal to obtain the optimized signal, which is regarded as the first layer of the IMF. Next, the remaining signals are calculated and used as a new signal. After the signal decomposition termination condition is met, the separation of the blood flow signal is completed. The specific flow to separate the blood flow signal by the optimized EMD algorithm is shown in Figure 1.

### 2.3. Separation Effect of Blood Flow and Tube Wall Signals by Doppler Ultrasound

In this study, the separation effects of blood flow and vessel wall signals by Doppler ultrasound are evaluated factored into the relative error of the average frequency curve of the blood flow signal (*E*_f_) and the mean relative error (MRE) of the power spectrum of the blood flow signal. They are commonly used blood flow evaluation parameters, expressed as follows:

(17) $$\begin{array}{c} E_{f}=\sqrt{\frac{\sum_{t}{\left[F\left(t\right)-F_{s}\left(t\right)\right]}^{2}}{\sum_{t}F_{s}{\left(t\right)}^{2}}}, \end{array}$$

where *F*_*s*_(*t*) is the average frequency of the standard pure blood flow signal and *F*(*t*) is the average frequency of the blood flow signal extracted by the separation algorithm.

(18) $$\begin{array}{c} \mathrm{MRE}=\frac{1}{M}\sum_{i=1}^{M}\frac{\sum_{f}\left|P_{1}\left(f,i\right)-P_{2}\left(f,i\right)\right|}{\sum_{f}P_{2}\left(f,i\right)}, \end{array}$$

where *f* represents the frequency; *M*is the total number of blood flow signal segments; and *P*_1_ and *P*_2_, respectively, represent the power spectral density of the initial blood flow signal and the separated blood flow signal in the *i*-th time period.

### 2.4. Treatment Plans and Observation Indicators for Different Groups

Patients in both groups were examined by the ultrasonic diagnostic instrument produced by Philips, and the probe frequency was adjusted to 2.0–5.0 mhz. At the beginning of the examination, the position of the umbilical cord was first determined, and the placental umbilical artery was sampled with a volume of 2 mL to ensure that the arterial vasculature angle and pulse Doppler sampling should be below 30°. Then, the blood flow spectrum was determined.

The control group was treated with magnesium sulfate alone, and the observation group was treated with Compound Danshen injection and magnesium sulfate. The specific scheme was as follows.

Control group used magnesium sulfate alone. The dosage of the drug was 4∼10 mL/time, mixed and diluted with 5% glucose injection and then intravenously infused, once a day on average, for a total of 10 days.

Observation group used Compound Danshen injection and magnesium sulfate. The dosage and method of magnesium sulfate were the same as the control group. The dosage of Compound Danshen injection was 10–20 mL/time, and it was diluted with 100–500 mL glucose injection with a concentration of 5%. Intravenous infusion was carried out once a day on average. The treatment also lasted 10 days.

The two groups were compared for the blood pressure and 24 h urine protein before and after treatment; the levels of serum endothelin-1 (ET-1), homocysteine (Hcy), and C-reactive protein (CRP) before and after treatment; the changes of blood coagulation function indexes, such as plasma prothrombin time (PT), fibrinogen (FIB), activated partial thromboplastin time (APTT), and plasma thrombin time (TT) before and after treatment; and the outcome of pregnancy.

### 2.5. Efficacy Evaluation

After the treatment, if the relevant clinical symptoms (such as edema, urine protein) basically disappear and the fetus is delivered smoothly, it is considered markedly effective; if the relevant clinical symptoms have been greatly reduced and, although the fetus can be delivered naturally, there are different degrees of suffocation, it is considered effective; if the relevant clinical symptoms of the patient are not significantly reduced and the pregnancy is forced to terminate, it is considered ineffective. Total effective rate = (markedly effective + effective)/number of cases × 100%.

### 2.6. Statistical Analysis

In this study, SPSS 22.0 was used for data processing and analysis, measurement data was expressed in the form of “$\bar{x}\pm s$,” and *t*-test was used for comparison between groups. Count data were expressed by “n” (%), and chi-square test was used for comparison between groups. *P* *<* 0.05 indicated that the difference was statistically significant.

## 3. Results

### 3.1. Separation Results of Blood Flow Signal Based on Optimized EMD Algorithm

**Figure 2:**
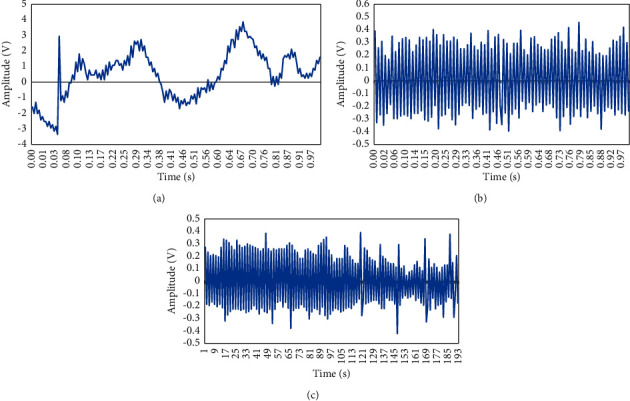
The separation effects of blood flow signal based on EMD optimization algorithm: (a) original blood flow signal diagram with tube wall; (b) blood flow signal separated by EMD algorithm; (c) blood flow signal separated by the optimized EMD algorithm.

The ultrasound Doppler blood flow signal was separated using the optimized EMD algorithm, and the result is shown in Figure 2. It was noted that the optimized EMD algorithm can separate the blood flow signal and the tube wall signal.

**Figure 3:**
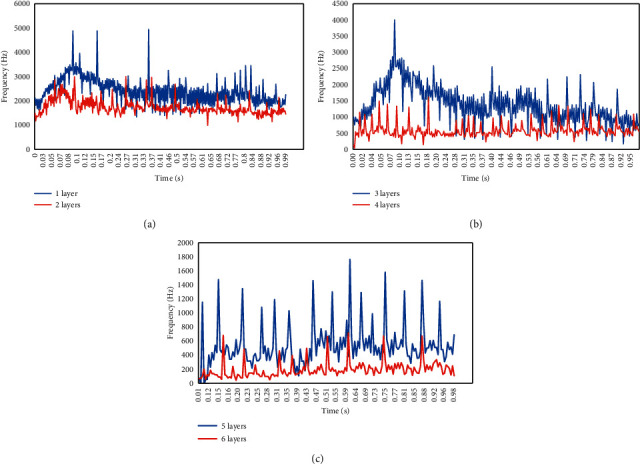
Multilayer instantaneous frequencies of blood flow signal decomposed by the optimized EMD algorithm: (a) 1∼2 layers; (b) 3∼4 layers; (c) 5∼6 layers.

The optimized EMD algorithm was used to decompose the ultrasound Doppler blood flow signal into multiple instantaneous frequencies. Here, the instantaneous frequencies of the 1st to 6th layers were analyzed (Figure 3). With the continuous increase of the number of layers, the instantaneous frequency corresponding to the IMF lowered continuously.

**Figure 4:**
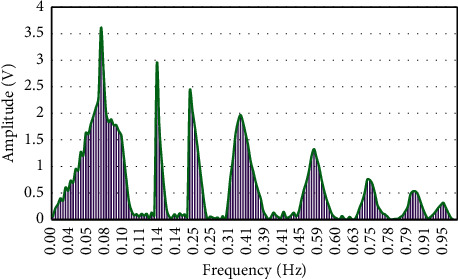
Changes of blood flow signal amplitude at different frequencies.

Then, the changes of instantaneous frequency over the amplitude were further analyzed (Figure 4). It was found that as the instantaneous frequency continued to increase, its corresponding amplitude gradually decreased.

**Figure 5:**
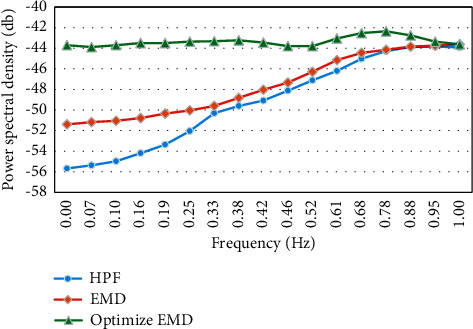
The power spectral density curves of blood flow signals separated by different algorithms.

The traditional EMD algorithm, the optimized EMD algorithm, and the high-pass filter method (HPF) were compared for the power spectral density after the ultrasound Doppler blood flow signal was separated (Figure 5). The power spectral density of blood signals separated by the optimized EMD algorithm was greater than those of the other two algorithms under different frequencies.

### 3.2. Separation Effects of Blood Flow and Tube Wall Signal by Optimized EMD Algorithm

**Figure 6:**
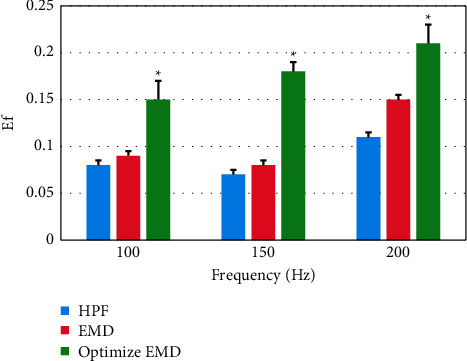
Comparison of Ef values of different algorithms under different frequencies (^∗^indicates statistical difference compared with traditional EMD algorithm).

**Figure 7:**
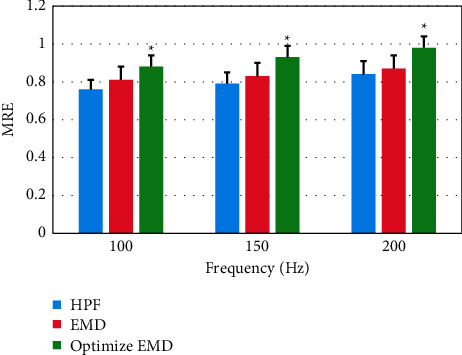
Comparison of MRE values of different algorithms under different frequencies (^∗^indicates statistical difference compared with traditional EMD algorithm).

Different algorithms were compared for the Ef and MRE values (Figures 6 and 7). It was found that the Ef and MRE values of the optimized EMD algorithm at different frequencies were significantly higher than those of the other two algorithms, and the optimized EMD algorithm showed statistically significant differences from the traditional EMD algorithm in Ef and MRE values (*P* *<* 0.05).

### 3.3. Clinical Efficacy

As for the total effective rate of the two groups of patients in this study, the total effective rate of the observation group was significantly better, and the difference compared with the control group was statistically significant (*P* *<* 0.05) (Table 1).

### 3.4. Blood Pressure

In this study, there was no significant difference in various indicators between the two groups of patients before treatment (*P* *>* 0.05). After treatment, from the perspective of blood pressure control, the observation group's SBP, DBP, and 24 h urine protein indexes were significantly better, and the difference was statistically significant compared with the control group (*P* *<* 0.05) (Table 2).

### 3.5. Expression of ET-1, CRP, and Hcy

In this study, there was no significant difference in the expression of ET-1, CRP, and Hcy between the two groups of patients before treatment (*P* *>* 0.05). After treatment, the ET-1, CRP, and Hcy indexes of the two groups of patients decreased significantly, and the decrease in the observation group was more obvious than that in the control group (*P* *<* 0.05) (Table 3).

### 3.6. Coagulation Function

Before treatment, there was no significant difference in the levels of PT, APTT, TT, and FIB between the two groups (*P* *>* 0.05); after treatment, the levels of PT, APTT, TT, and FIB in the two groups were better than those before treatment, and the observation group was better than the control group. The difference was statistically significant (*P* *<* 0.05) (Table 4).

### 3.7. Maternal and Infant Outcome

As shown in Table 5, the incidence of adverse maternal and infant outcomes in the observation group was significantly lower than that in the control group (*P* *<* 0.05).

## 4. Discussion

Compound Danshen injection can prevent coagulation dysfunction, cerebrovascular accidents, and other complications. Combined with magnesium sulfate, it can not only effectively alleviate PIH syndrome, but also improve blood circulation and immunity [13, 14]. In this study, it was found that the blood pressure and 24 h urine protein of the observation group after treatment were significantly better than those in the control group (*P* *<* 0.05). It can be inferred that the treatment plan of Compound Danshen injection combined with magnesium sulfate is helpful to optimize the blood pressure control of patients with PIH syndrome. In addition, the total effective rate in the observation group was significantly higher than that of the control group (*P* *<* 0.05). It can be inferred that the treatment plan of Compound Danshen injection combined with magnesium sulfate can improve the maternal and infant outcomes.

Hcy is an important intermediate product in the process of methionine metabolism. The increase of serum Hcy level in pregnant women is closely related to the occurrence and development of HDP [15]. CRP is one of the important indicators that reflect the body's inflammatory response. The increase in serum CRP concentration in HDP patients has a certain correlation with vascular endothelial damage [16]. The results of this study showed that, after treatment, the serum Hcy and CRP levels of the observation group were significantly lower than those of the control group, suggesting that the combination of Compound Danshen injection and magnesium sulfate can significantly reduce the serum Hcy and CRP levels of HDP patients.

From the perspective of traditional Chinese medicine, hypertension during pregnancy belongs to the category of “blood stasis,” and its occurrence is related to the blood stasis, deficiency of qi and blood, and obstruction of blood circulation. Therefore, the treatment aims to promote blood circulation, remove blood stasis, and dredge the meridians. Compound Danshen injection is a Chinese patent medicine composed of traditional Chinese medicine Danshen and Jiangxiang. Danshen is slightly cold and bitter, and it has the effects of calming the mind, removing blood stasis, and promoting blood circulation [17, 18]. Jiangxiang is known for nourishing the heart, dredging the meridians, relieving pain, stopping bleeding, and removing blood stasis [19]. The combination of the two has the effect of calming the mind, promoting blood circulation, and removing blood stasis [20]. It has been found that the polyphenols in Danshen can inhibit the release of a variety of coagulation factors [21]. By inhibiting the release of the *α* receptor, the aggregation of platelets is greatly reduced, so as to relax the capillaries and increase the secretion of nitric oxide (NO) in endothelial cells, to reduce the oxygen consumption of the myocardium, improve microcirculation, and effectively control the blood pressure [22]. At the same time, Danshen can positively affect the blood rheology and increase the perfusion flow of the placenta, so that the placenta can get sufficient blood supply and the occurrence of adverse pregnancy outcomes is greatly reduced [23]. Dalbergin and volatile oil in Jiangxiang can regulate the balance between ET and NO and antagonize thrombosis [24, 25], thereby improving the patient's oxidative stress and blood hypercoagulability and reducing the incidence of adverse pregnancy outcomes.

In conclusion, the treatment of PIH syndrome patients with Compound Danshen injection combined with magnesium sulfate proved to be effective and can improve maternal and infant outcomes; control blood pressure; and reduce 24 h urine protein and serum ET-1, Hcy, and CRP. Additionally, it can improve the blood coagulation function, and thus the treatment plan can be widely promoted in the clinic.

## 5. Conclusion

Based on the EMD algorithm, this study established a separation method of ultrasound blood flow signal, which was then applied to the diagnosis of patients with pregnancy-induced hypertension syndrome. Compound Danshen injection combined with magnesium sulfate was used to treat patients with pregnancy-induced hypertension syndrome. EMD algorithm can improve the accuracy of blood flow signal separation in ultrasonic images. Treatment of pregnancy-induced hypertension syndrome by Compound Danshen injection combined with magnesium sulfate can improve maternal and infant outcomes; control blood pressure; reduce 24 h urinary protein and serum ET-1, Hcy, and CRP levels; and improve coagulation function. However, the study still has the following disadvantages. It only analyzes the strain curve of the ultrasonic imaging blood flow signal. In future work, on the basis of ultrasonic imaging blood flow signal strain curve, the peak strain, negative peak strain, and positive peak strain rate should be evaluated, to evaluate the value of blood flow signal decomposition based on EMD algorithm in the diagnosis of pregnancy-induced hypertension syndrome. In conclusion, the EMD algorithm optimized in this study is of great significance for the diagnosis based on ultrasonic blood flow signals. Compound Danshen injection combined with magnesium sulfate can improve clinical indicators of pregnancy-induced hypertension syndrome, providing a reference basis for the clinical diagnosis and treatment of pregnancy-induced hypertension syndrome.

## Figures and Tables

**Table 1:** Comparison of the total effective rate of the two groups of patients after treatment.

Index	Observation group	Control group	*P* value
Markedly effective (*n*)	23	18	0.031^*∗*^
Effective (*n*)	14	11	0.048^*∗*^
Ineffective (*n*)	3	11	0.003^*∗∗*^
Total effective rate (%)	92.50	72.50	0.016^*∗*^

^∗^represents a statistical difference compared with the control group, *P* *<* 0.05; ^*∗∗*^represents a significant difference compared with the control group, *P* *<* 0.01.

**Table 2:** Comparison of blood pressure control between the two groups of patients after treatment.

Index	Time	Observation group	Control group	*P* value
SBP (mmHg)	Before treatment	158.32 ± 11.86	159.33 ± 11.90	0.913
	After treatment	127.85 ± 9.34	138.49 ± 9.11	0.021^*∗*^

DBP (mmHg)	Before treatment	117.09 ± 11.56	119.13 ± 10.97	0.861
	After treatment	78.35 ± 9.77	85.84 ± 8.71	0.032^*∗*^

24 h urine protein (g)	Before treatment	4.19 ± 1.36	4.17 ± 1.29	0.953
	After treatment	1.36 ± 0.24	2.25 ± 0.26	0.016^*∗*^

^∗^represents a statistical difference compared with the control group, *P* *<* 0.05.

**Table 3:** The expression of CRP and Hcy in the two groups of patients before and after treatment.

Index	Time	Observation group	Control group	*P* value
ET-1 (mg/L)	Before treatment	52.41 ± 5.73	52.97 ± 6.04	0.864
	After treatment	43.11 ± 4.28	49.88 ± 5.03	0.023^*∗*^

CRP (mg/L)	Before treatment	16.24 ± 1.87	16.28 ± 1.93	0.904
	After treatment	3.61 ± 0.54	9.28 ± 0.93	0.008^*∗∗*^

Hcy (µmol/L)	Before treatment	23.11 ± 2.97	23.24 ± 3.05	0.895
	After treatment	12.56 ± 1.74	19.15 ± 1.86	0.009^*∗∗*^

^∗^represents a statistical difference compared with the control group, *P* *<* 0.05; ^*∗∗*^represents a significant difference compared with the control group, *P* *<* 0.01.

**Table 4:** The coagulation function indexes of the two groups before and after treatment.

Index	Time	Observation group	Control group	*P* value
PT (s)	Before treatment	10.16 ± 1.01	10.18 ± 0.89	0.693
	After treatment	12.98 ± 1.25	11.01 ± 0.94	0.041

APTT (s)	Before treatment	21.41 ± 2.15	21.36 ± 2.21	0.572
	After treatment	29.85 ± 2.43	25.07 ± 2.11	0.032^*∗*^

TT (s)	Before treatment	16.12 ± 0.49	16.16 ± 0.55	0.610
	After treatment	17.85 ± 0.27	16.86 ± 0.28	0.038^*∗*^

FIB (g/L)	Before treatment	5.06 ± 0.46	5.04 ± 0.47	0.566
	After treatment	3.12 ± 0.52	4.32 ± 0.64	0.021^*∗*^

^∗^represents a statistical difference compared with the control group, *P* *<* 0.05.

**Table 5:** The incidence of adverse maternal and infant outcomes in the two groups.

Index	Observation group	Control group	*P* value
Premature delivery (n)	1	2	-
Placental abruption (n)	0	1	
Eclampsia (n)	0	1	
Postpartum hemorrhage (n)	1	2	
Weak contractions (n)	0	1	
Neonatal asphyxia (n)	1	2	
Incidence (n (%))	3 [7.50]	9 [22.50]	0.017^*∗*^

^∗^represents a statistical difference compared with the control group, *P* *<* 0.05.

## Data Availability

The data used to support the findings of this study are available from the corresponding author upon request.
